# Reduced vascular risk factors in Parkinson's disease dementia and dementia with Lewy bodies compared to Alzheimer's disease

**DOI:** 10.1002/brb3.916

**Published:** 2018-02-05

**Authors:** Po‐Chi Chan, Cheng‐Yu Wei, Guang‐Uei Hung, Pai‐Yi Chiu

**Affiliations:** ^1^ Department of Neurology Show Chwan Memorial Hospital Changhua Taiwan; ^2^ Department of Neurology Chang Bing Show Chwan Memorial Hospital Changhua Taiwan; ^3^ Department of Nuclear Medicine Chang Bing Show Chwan Memorial Hospital Changhua Taiwan

**Keywords:** Alzheimer's disease, dementia with Lewy bodies, Lewy body dementia, Parkinson's disease dementia, vascular risk factors

## Abstract

**Objectives:**

The association of vascular risk factors (VRFs) with incidence of Alzheimer's disease (AD) and vascular dementia (VaD) has been well studied. However, the association between VRFs and non‐AD dementia is seldom investigated. In this study, we aim to compare the concurrence of VRFs of Lewy body dementia (LBD) to AD.

**Materials & Methods:**

We consecutively enrolled patients with dementia with Lewy bodies (DLB), Parkinson's disease dementia (PDD), and AD, and the prevalence of arterial hypertension, diabetes mellitus, hypercholesterolemia, hyperlipidemia, smoking, and obesity was assessed and compared.

**Results:**

A total of 167 consecutive patients were studied. Among them, 55 had DLB, 34 had PDD, and 78 had AD. History of any VRF among three groups was not significantly different. In addition, the patients with AD had significantly higher systolic pressure (SBP), diastolic pressure (DBP), waist, body mass index (BMI), ac glucose, and HbA1c (all *p*‐value < .005). After a stepwise procedure that considered age, sex, disease severity, antiparkinson drugs, systolic and diastolic blood pressures, glycated hemoglobin, body mass index, triglyceride, total cholesterol, and low‐density lipoprotein, SBP in the third tertile (144–198 mmHg), BMI in the second tertile (23.6–26.4), and TG in the third tertile (127–310) were significantly lower in LBD than in AD.

**Conclusions:**

The VRFs are less associated with LBD than with AD. DLB and PDD showed a similar pattern of the association of VRFs. SBP, BMI, and TG were significantly lower in LBD compared with AD.

## 1 INTRODUCTION

Growing evidence has supported the influence of vascular risk factors (VRFs) on incidence of dementia due to Alzheimer's disease (AD) as well as of vascular dementia (VaD) (Biessels & Kappelle, 2005; Fitzpatrick et al., 2009; Forette et al., 2002; Gorelick, 2004; Gudala, Bansal, Schifano, & Bhansali, 2013; Kivipelto et al., 2005; Launer et al., 2000; Luchsinger, Tang, Stern, Shea, & Mayeux, 2001; Luchsinger et al., 2005; Reitz et al., 2010). A recent meta‐analysis summarized the risk of dementia associated with diabetes and reported a 73% increased risk of all dementia, a 56% increase in AD, and an 127% increase in VaD (Gudala et al., 2013). Midlife VRFs including hypertension, hypercholesterol, obesity, and diabetes are most contributing to late‐life incident dementia due to AD (Biessels & Kappelle, 2005; Fitzpatrick et al., 2009; Kivipelto et al., 2005; Luchsinger et al., 2005; Reitz et al., 2010). Besides, most of the VRFs including hypertension, diabetes, heart disease, dyslipidemia, and smoking are contributing to the incident dementia associated with stroke (Forette et al., 2002; Gorelick, 2004; Launer et al., 2000; Luchsinger et al., 2001). However, studies on the association between VRFs and Lewy body dementia (LBD) are limited. Previous studies had put more emphasis on the association of VRFs mainly with Parkinson's disease (PD) patients regardless of whether or not they developed dementia, and the results were controversial (Haugarvoll, Aarsland, Wentzel‐Larsen, & Larsen, 2005; Papapetropoulos, Villar, & Mash, 2006; Papapetropoulos et al., 2004; Rektor et al., 2009; Schelp, Mendes‐Chiloff, Bazan, Paduan, & Pioltini, 2012; Scigliano et al., 2006; Simon, Chen, Schwarzschild, & Ascherio, 2007; Sławek et al., 2008, 2010; Toledo et al., 2013). Reduced risk factors for vascular disorders were found in patients with PD (Scigliano et al., 2006) or with α‐synucleinopathy (Toledo et al., 2013). Some studies suggested that PD risk was not significantly related to history of hypertension, hypercholesterolemia, or diabetes (Haugarvoll et al., 2005; Schelp et al., 2012; Simon et al., 2007; Sławek et al., 2008, 2010), and other studies showed positive contribution (Papapetropoulos et al., 2004, 2006; Rektor et al., 2009). Although dementia with Lewy bodies (DLB) is the second most common degenerative dementia (Karantzoulis & Galvin, 2013; McKeith et al., 1996), the study of VRFs associated with DLB is scarce (Boot et al., 2013). On account of findings on the association of VRFs in Parkinson's disease dementia (PDD) and DLB are still limited, we aim to study and compare the prevalence of various VRFs of DLB and PDD with AD. We propose that DLB and PDD will present with very approximate patterns of association of VRFs because of their similar clinical and pathological manifestation, but association patterns between DLB/PDD and AD will be different.

## 2 METHODS

### 2.1 Participants

This study is part of a dementia project in central Taiwan (Chiu, Tsai, Chen, Liu, & Lai, 2015). A consecutive series of patients visited the dementia clinic fulfill the criteria for AD, DLB, or PDD were enrolled, and their demographic and clinical data including VRFs were analyzed in this study. The dementia patients were diagnosed according to the criteria for primary degenerative dementia in the fourth edition of the Diagnostic and Statistic Manual of Mental Disorders (DSM‐IV). The DLB patients were diagnosed according to the revised consensus criteria for probable or possible DLB developed by the third report of the DLB consortium (McKeith et al., 2005). The PDD patients were diagnosed according to the clinical criteria for possible or probable PDD developed by the Movement Disorder Society (MDS) in 2007 (Emre et al., 2007). The patients with AD were diagnosed according to the criteria for probable or possible AD developed by the National Institute of Neurologic and Communicative Disorders and Stroke and the Alzheimer's Disease and Related Disorders Association (NINCDS‐ADRDA) (McKhann et al., 1984).

### 2.2 Assessment tools

The following variables in all participants were analyzed: smoking, diabetes, hypertension, cerebrovascular disease, cardiovascular disease, and blood pressure, sugar, cholesterol, triglycerides, and total lipids. Smoking, diabetes, hypertension, cerebrovascular, and cardiovascular diseases were analyzed as dichotomous variables with no history or not present as reference categories. Blood pressure, blood sugar, cholesterol, triglycerides, and total lipids were categorized into tertiles based on their distribution in controls, with lowest tertile as reference. Patients with diabetes or hypertension were assigned to the third tertile of blood sugar or blood pressure (systolic and diastolic), respectively.

The global severity of dementia is assessed according to the Clinical Dementia Rating (CDR) scale and sum of boxes of CDR (CDR‐SB) (Morris, 1993). Cognitive functions were assessed with the Mini‐mental State Examination (MMSE) (Folstein, Folstein, & McHugh, 1975) and the Cognitive Abilities Screening Instrument (CASI) (Teng et al., 1994). Motor functions were assessed with motor score of the Unified Parkinson's Disease Rating Scale (UPDRS‐m) (Ballard et al., 1997). Fluctuation was diagnosed when a clinical history of fluctuation in cognition and a Mayo Fluctuations Composite Score (MFCS) > 2 were both present (Ferman et al., 2004). Visual hallucinations (VHs) were diagnosed when a clinical history of recurrent VHs and a hallucination subscale in NPI (NPH‐H) > 0 were both present (Cummings et al., 1994). Parkinsonism was diagnosed when at least 2 of the following were present: bradykinesia, tremor, rigidity, and postural instability. REM sleep behavior disorder (RBD) was diagnosed when the minimal criteria for RBD in the International Classification of Sleep Disorders (ICSD)‐2 were positive or atonia was accompanied by REM sleep during polysomnography (PSG) study (American Academy of Sleep Medicine, 2005). Cognitive tests of all patients and the activities of daily living function assessments according to their main caregiver were performed by a trained neuropsychologist. Dementia and subtype of dementia were made by a consensus meeting composed of three senile neurologists, one geriatric psychiatrist, and one neuropsychologist. All patients received at least a cerebral CT or MRI, and most patients received a set of blood screening tests including complete blood count, COT, GPT, BUN, creatinine, total cholesterol, TG, ac glucose, and HbA1c.

### 2.3 Data analysis

The Chinese version of SPSS 19.0 for Windows (IBM, SPSS Inc., Chicago) was used for statistical analyses. Comparisons between DLB, PDD, and AD groups on demographic data, neuropsychological tests, motor score of UPDRS, VHs subscale (frequency x severity) of NPI, the composite scores (frequency x severity) of NPI, and measurements of VRFs were analyzed using one‐way ANOVA with either the Bonferroni or Dunnett T3 post hoc analysis according to the homogeneity of variances. Sex, CDR, clinical features, clinical history of VRFs, and current medications were analyzed with the chi‐square test. For the comparison of association of VRFs among patients with LBD and AD, we used a multivariable estimate model, and odds ratios (ORs) were adjusted for age, sex, smoking, systolic and diastolic blood pressures, fasting glucose, body mass index, triglyceride, and total cholesterol.

### 2.4 Ethical consideration

The Committee for Medical Research Ethics of Show Chwan Hospital reviewed the project, and the Data Inspectorate approved it. All participants signed the informed consent when they agreed to join the study.

## 3 RESULTS

A total of 167 patients with complete data were analyzed. Among them, 55 had DLB, 34 had PDD, and 78 had AD. Table 1 summarizes the demographic characteristic of all patients. DLB group had significant higher CDR‐SB (*F* = 4.045, *p* = .018) than AD. Both DLB and PDD groups had higher UPDRS motor score than AD. Sex, age, onset age, education, CDR, MMSE, and CASI were not different between three groups of patients (*p* = NS). Comparisons of clinical features showed higher frequencies of fluctuation, visual hallucinations (VHs), parkinsonism, and RBD for DLB group than AD group (all *p* < .05). PDD group had significantly higher frequencies of fluctuation, parkinsonism, VHs, and RBD than the AD group (all *p* < .05).

**Table 1:** Demographic and background characteristics of DLB, PDD, and AD patients

	DLB	PDD	AD	*F*/χ2	*p*‐value
*N*	55	34	78		
Sex, m/f	33/22	15/19	41/37	2.161	NS
Education (year)	6.7 ± 5.5	6.4 ± 4.4	6.0 ± 5.2	0.288	NS
Age (year)	78.4 ± 7.1	75.5 ± 9.6	77.6 ± 8.3	1.282	NS
Onset age (year)	76.4 ± 7.0	72.9 ± 9.5	74.3 ± 8.0	2.287	NS
CDR 0.5/1/2/3	16/32/7/0	9/21/4/0	19/38/19/2	6.844	NS
CDR‐SB	6.3 ± 3.0	5.4 ± 2.7	6.5 ± 3.1	1.693	NS
MMSE	19.7 ± 6.6	21.3 ± 5.7	18.1 ± 7.1	2.876	NS
CASI	63.0 ± 20.7	69.1 ± 17.9	58.1 ± 23.4	3.204	.043
NPI	22.6 ± 12.7	15.9 ± 9.4	19.5 ± 14.9	2.655	NS
NPI‐H	1.9 ± 2.5	0.7 ± 1.6	0.8 ± 1.7	5.902	.003
UPDRS‐m	13.6 ± 7.8	16.7 ± 8.8	7.4 ± 6.6	21.942	<.001
Clinical features					
Fluctuation, *n* (%)	19 (34.5)	7 (20.6)	10 (12.8)	9.027	.011
VHs, *n* (%)	25 (45.5)	7 (20.6)	17 (21.8)	10.287	.006
Parkinsonism, *n* (%)	47 (85.5)	34 (100)	14 (17.9)	92.289	<.001
RBD, *n* (%)	23 (41.8)	11 (32.4)	6 (7.7)	22.277	<.001

DLB, dementia with Lewy bodies; PDD, Parkinson's disease dementia; AD, Alzheimer's disease; Onset age, onset age of dementia; CDR, Clinical Dementia Rating scale; CDR‐SB, sum of boxes of CDR; MMSE, Mini‐mental State Examination; CASI, Cognitive Abilities Screening Instrument; NPI, total score of 12‐domain Neuropsychiatric Inventory; NPI‐H, subscore of hallucinations in NPI; UPDRS‐m, motor score of the Unified Parkinson's Disease Rating Scale. VHs, visual hallucinations; RBD, REM sleep behavior disorder.

The comparisons of the clinical history and medication are summarized in Table 2. The results showed that history of hypertension and diabetes was lower in PDD than AD [odds ratio (OR) = 0.38]. Atherosclerosis was lower in DLB than AD (OR = 0.26).

**Table 2:** Comparison of history of vascular risk factors (VRFs) and current medication among DLB, PDD, and AD groups

	*n* (%)			Odds ratio (95% CI)		
	DLB	PDD	AD	DLB versus AD	PDD versus AD	PDD versus DLB
History						
Hypertension	33 (60.0)	16 (47.1)	51 (65.4)	0.79 (0.39–1.62)	0.47 (0.21–1.07)	0.59 (0.25–1.40)
Diabetes	16 (29.1)	6 (17.6)	24 (30.8)	0.92 (0.43–1.96)	0.48 (0.18–1.32)	0.52 (0.18–1.50)
Smoking	22 (40.0)	8 (25.8)	23 (29.5)	1.59 (0.77–3.90)	0.83 (0.33–2.13)	0.52 (0.20–1.37)
Physical inactivity	36 (65.5)	17 (54.8)	48 (61.5)	1.18 (0.58–2.43)	0.76 (0.33–1.76)	0.64 (0.26–1.58)
Hyperlipidemia	4 (7.3)	2 (5.9)	11 (14.1)	0.48 (0.14–1.59)	0.38 (0.08–1.82)	0.80 (0.14–4.60)
Atherosclerosis	2 (3.6)	2 (5.9)	9 (11.5)	0.29 (0.06–1.40)	0.48 (0.10–2.35)	1.25 (0.46–3.37)
Current medication						
Antihypertensive drugs	22 (40.0)	15 (44.1)	37 (47.4)	0.74 (0.37–1.49)	0.88 (0.39–1.97)	1.18 (0.50–1.81)
Antidiabetic drugs	11 (20.0)	3 (8.8)	21 (26.9)	0.68 (0.30–1.55)	0.26 (0.07–0.95)^a^	0.39 (0.10–1.50)
Statins	3 (5.5)	2 (5.9)	6 (7.7)	0.69 (0.17–2.90)	0.75 (0.14–3.92)	1.08 (0.17–6.84)
Antiplatelet drugs	16 (29.1)	7 (20.6)	17 (21.8)	1.47 (0.67–3.25)	0.93 (0.35–2.50)	0.63 (0.23–1.74)
Antiparkinson drugs	26 (47.3)	27 (79.4)	10 (12.8)	6.10 (2.61–14.25)^b^	26.23 (9.05–75.99)^b^	4.30 (1.61–11.53)^b^
Antidementia drugs	5 (9.1)	10 (29.4)	19 (24.4)	0.31 (0.11–0.89)^a^	1.29 (0.53–3.19)	4.17 (1.28–13.54)^b^

*n*, Number of cases; DLB, dementia with Lewy bodies; PDD, Parkinson's disease dementia; AD, Alzheimer's disease.

a *p *<* *.05.

b *p *<* *.005.

The measurements of VRFs are summarized in Table 3 and show significantly higher systolic pressure (SBP; *F* = 14.390, *p* < .001), diastolic pressure (DBP; *F* = 3.173, *p* = .043), waist (*F* = 8.791, *p* < .001), body mass index (BMI; *F* = 15.009, *p* < .001), ac glucose (*F* = 3.514, *p* = .032), and HbA1c (*F* = 8.167, *p* < .001) in AD than other two groups.

**Table 3:** Comparison of measurement of vascular risk factors (VRFs) among DLB, PDD, and AD groups

	DLB (*n* = 55)		PDD (*n* = 34)		AD (*n* = 78)		*F*	*p*	post hoc
	Mean	SD	Mean	SD	Mean	SD			
Systolic BP, mmHg	130.4	16.9	131.8	24.4	143.1	19.7	7.914	.001	DLB = PDD; DLB < AD; PDD < AD
Diastolic BP, mmHg	71.0	10.5	68.9	11.4	73.9	10.7	2.770	.066	DLB = PDD; DLB = AD; PDD = AD
Heart rate, beats/min	75.3	14.2	74.7	16.6	76.5	14.4	0.206	.814	DLB = PDD; DLB = AD; PDD = AD
BMI, kg/m^2^	23.3	3.1	24.9	3.2	25.7	2.9	10.156	<.001	DLB < AD; DLB = PDD; PDD = AD
Blood ac glucose, mg/dl	116.4	40.0	101.2	52.6	125.7	44.0	3.400	.036	PDD < AD; DLB = PDD; DLB = AD
HbA1c	6.8	0.9	6.7	0.8	7.6	1.3	12.210	<.001	DLB = PDD; DLB < AD; PDD < AD
TG	93.4	27.3	101.2	52.6	139.3	55.1	17.369	<.001	DLB = PDD; DLB < AD; PDD < AD
Total cholesterol, mg/dl	174.0	37.9	173.6	30.5	175.7	37.9	0.058	.944	DLB = PDD; DLB = AD; PDD = AD
LDL‐C, mg/dl	107.5	30.5	104.7	28.2	113.1	34.0	0.975	.380	DLB = PDD; DLB = AD; PDD = AD

*n*, number of cases; DLB, dementia with Lewy bodies; PDD, Parkinson's disease dementia; AD, Alzheimer's disease; BP, blood pressure; BMI, body mass index; HbA1c, glycated hemoglobin; LDL‐C, low‐density lipoprotein; HDL‐C, high‐density lipoprotein.

The comparisons of VRFs between LBD (combined DLB and PDD) and AD are summarized in Table 4. Multivariable ORs were adjusted for age, sex, smoking, systolic and diastolic blood pressures, fasting glucose, body mass index, triglyceride, and total cholesterol, and showed that SBP in the first tertile (80‐128 mmHg; OR = 5.4), BMI in the first tertile (14.1‐23.5; OR = 3.1) and the second tertile (23.6‐26.4; OR = 4.0), and TG in the first tertile (23‐90; OR = 17.9) were significantly higher in LBD than in AD. Total cholesterol was lower in LBD in the first tertile (67‐154; OR = 0.3) than AD.

**Table 4:** Multivariable risk estimates (ORs) for LBD (DLB + PDD) patients compared to AD patients

	*n* (%)		Crude OR^a^ (95% CI)	Multivariable OR^b^ (95%CI)
	AD (*n* = 78)	LBD (*n* = 89)		
Antiparkinson drugs	10 (12.8)	53 (59.6)	10.01 (4.56–22.00) *p* < .001	c
SBP, mmHg				
1st tertile (87–128)	16 (20.5)	39 (43.8)	1	1
2nd tertile (129–143)	25 (31.1)	29 (32.6)	0.41 (0.13–1.27)	0.54 (0.20–1.45)
3rd tertile (144–198)	37 (47.4)	21 (23.6)	0.20 (0.06–0.71) *p *= 013	0.28 (0.10‐0.76) *p* = .012
DBP, mmHg				
1st tertile (48–66)	23 (23.8)	30 (33.7)	1	c
2nd tertile (67–76)	23 (38.9)	32 (36.0)	2.21 (0.67–7.30)	c
3rd tertile (77–98)	32 (37.3)	27 (30.3)	1.83 (0.54–6.22)	c
Fasting glucose, mg/dl				
1st tertile (69–102)	20 (25.6)	34 (38.2)	1	c
2nd tertile (103–118)	22 (28.2)	34 (38.2)	1.57 (0.52–4.70)	c
3rd tertile (119–349)	36 (46.2)	21 (23.6)	0.64 (0.19–2.11)	c
HbA1c, mmol/mol				
1st tertile (4.2–6.7)	17 (21.8)	40 (44.9)	1	1
2nd tertile (6.8–7.2)	17 (21.8)	39 (43.8)	0.68 (0.24–1.95)	0.72 (0.28–1.84)
3rd tertile (7.3–11.6)	44 (56.4)	10 (11.2)	0.19 (0.06–0.65) *p *= .008	0.15 (0.05–0.41) *p *< .001
BMI, kg/m^2^				
1st tertile (15.6–23.5)	13 (16.7)	43 (48.3)	1	1
2nd tertile (23.6–26.4)	32 (41.0)	24 (27.0)	0.20 (0.06–0.62) *p *= .006	0.29 (0.11–0.76) *p *= .012
3rd tertile (26.5–32.2)	33 (42.3)	22 (24.7)	0.34 (0.11–1.06)	0.42 (0.15–1.16)
TG, mg/dl				
1st tertile (23–90)	13 (16.7)	41 (46.7)	1	1
2nd tertile (91–126)	21 (26.9)	38 (42.1)	0.63 (0.22–1.82)	0.62 (0.25–1.58)
3rd tertile (127–310)	44 (56.4)	10 (11.2)	0.08 (0.02–0.30) *p *< .001	0.13 (0.04–0.36) *p *< .001
Total cholesterol, mg/dl				
1st tertile (104–154)	27 (34.6)	26 (29.2)	1	c
2nd tertile (155–189)	28 (35.9)	34 (38.2)	2.47 (0.71–8.63)	c
3rd tertile (191–282)	23 (29.5)	29 (32.6)	13.64 (2.10–88.71) *p *= .006	c
LDL, mg/dl				
1st tertile (37–95)	26 (33.3)	29 (32.6)	1	c
2nd tertile (96–121)	24 (30.8)	35 (35.8)	0.76 (0.21–2.75)	c
3rd tertile (122–203)	28 (35.9)	25 (28.1)	0.20 (0.01–1.18)	c

*n*, number of cases; AD, Alzheimer's disease; LBD, Lewy body dementia; DLB, dementia with Lewy bodies; PDD, Parkinson's disease dementia; HbA1c, glycated hemoglobin; BMI, body mass index; TG, triglyceride; LDL, low‐density lipoprotein.

a Crude ORs are adjusted for age and sex.

b Multivariable ORs are derived from a stepwise procedure that considered age, sex, disease severity, antiparkinson drugs, systolic and diastolic blood pressures, glycated hemoglobin, body mass index, triglyceride, total cholesterol, and low‐density lipoprotein.

c ORs absent for variables not included in multivariable regression analysis.

## 4 DISCUSSION

The main finding of this study was that the VRFs associated with DLB and PDD were similar but were distinct from those with AD. By comparing the clinical history of VRFs among three groups, there is no different association among DLB, PDD, and AD. This finding was partially consistent with the results of two recent studies regarding the risk factors for Lewy body disease (Boot et al., 2013; Sławek et al., 2010). However, the measurements of these factors in our study further demonstrated a different association pattern of VRFs between DLB/PDD and AD. DLB and PDD presented similar pattern and showed lower association with some of the factors compared to AD. This finding is consistent with two previous studies (Scigliano et al., 2006; Sławek et al., 2010; Toledo et al., 2013). Results from a large case–control study showed reduced risk factors for vascular disorders were found in patients with PD (Scigliano et al., 2006). A large‐scale study of the contribution of cerebrovascular disease in autopsy‐confirmed neurodegenerative disease cases revealed that the prevalence of vascular pathology and cerebrovascular disease was reduced in patients with α‐synucleinopathy compared to those with Alzheimer's disease (AD) (Toledo et al., 2013). Among the few researches regarding VRFs as well as metabolic syndrome (MetS), association with Lewy body disease revealed that reduced VRFs were noted in patients with PD (Sławek et al., 2010). Results of this study also revealed a modest decline of the risk with increasing blood cholesterol levels (Sławek et al., 2010).

Whether or not systemic or brain vascular diseases contribute to the progression of PD patients to the development of cognitive dysfunction or dementia is still controversial. Some studies of the association of VRFs with PD revealed no significantly contribution of VRFs to the risk of PD or to the progression of PD to PDD (Haugarvoll et al., 2005; Schelp et al., 2012; Simon et al., 2007; Sławek et al., 2008, 2010). The others provided positive conclusion (Papapetropoulos et al., 2004, 2006; Rektor et al., 2009). A prospective study of Parkinson's disease risk by Simon et al. on 2007 revealed no significantly relationship to history of hypertension, hypercholesterolemia, or diabetes, and the risk may modestly decline with increasing blood cholesterol levels (Simon et al., 2007). Haugarvoll et al. studied a large and representative cohort of patients with PD and found cerebrovascular risk factors were not associated with incident dementia (Haugarvoll et al., 2005). Sławek et al. on 2008 studied VRFs and white matter vascular abnormalities and revealed no significant contribution to cognitive impairment in patients with PD (Sławek et al., 2008, 2010). Schelp et al. studied the relationship between metabolic disorders and progression of dementia in PD and revealed that dementia in PD is age‐dependent and not related to disease duration (Schelp et al., 2012). Rektor et al. on 2009 studied the impact of brain vascular pathology on the clinical status of PD and found subclinical vascular pathology could influence the clinical status by contributing to motor and cognitive dysfunction in PD (Rektor et al., 2009). Papapetropoulos et al. on 2004 and 2006 studied the contribution of systemic or brain vascular diseases to the progression of PD and used very strict criteria to avoid the contamination of vascular parkinsonism, and their findings clearly suggested that the presence of vascular disease on idiopathic PD patients may aggravate PD severity (Papapetropoulos et al., 2004) and dementia (Papapetropoulos et al., 2006).

Leaving aside the controversy of contribution of brain or systemic vascular disorders to parkinsonism or dementia, well control of each VRF is important for the prevention of cardiovascular disorders. In older patients and those with significant comorbid illnesses, target of control of each VRF is probably different from that of younger or healthy adults (Huang & Davis, 2015). Although midlife VRFs are most contributing to late‐life incident dementia due to AD or other dementia (Biessels & Kappelle, 2005; Fitzpatrick et al., 2009; Kivipelto et al., 2005; Luchsinger et al., 2005; Reitz et al., 2010), the lower the better strategy should be utilized very cautiously when it is applied to older patients and those with significant functional limitation such as dementia and parkinsonism. Looser but reasonable targets may prevent the deterioration of cognitive function as well as reduce the incident dementia (Huang & Davis, 2015). Balance between the prevention of cardiovascular and cerebrovascular diseases and the preservation of cognitive functions deserves more attention when we are considering the target of control of each VRF associated with older patients. Determination of the most suitable targets of control needs more evidence from further researches of the elderly.

The study has limitations that should be noted. First, the sample size of PDD group in this study is relatively small. Further researches should include a greater number of PDD patients for a better comparison. Second, our research was conducted in only one hospital in central Taiwan. Therefore, selection bias may arise, and our findings may not be generalizable to all patients with DLB, PDD, or AD. Future studies should target recruitment of dementia patients from multiple centers in order to reduce the selection bias. Third, the comparison of VRFs between DLB, PDD, and AD in our study was cross‐sectional. Therefore, causal relationship of VRFs and dementia is not able to be speculated. Fourth, because of the lack of more determinative equipment or facilities, such as genetic study, dopamine transporter uptake imaging, amyloid plaque imaging, CSF biomarkers, or pathological studies, the diagnosis of DLB, PDD, and AD was based only on clinical criteria. Therefore, diagnosis bias may arise. Future studies should include longitudinal as well as pathological data in order to investigate the contribution of VRFs to LBD.

## AUTHOR'S CONTRIBUTION

P.C. Chan undertook the literature searches and the data analyses, edited all author contributions, and was mainly responsible for drafts of the manuscript. P.Y. Chiu undertook the data analyses and was responsible for revisions and drafts of the manuscript. C.Y. Wei participated in data analysis and contributed to revisions of the manuscript. G.U. Huang participated in data analysis and contributed to revisions of the manuscript.

## CONFLICT OF INTEREST

None declared.
